# Past Visits Present: TCF/LEFs Partner with ATFs for β-Catenin–Independent Activity

**DOI:** 10.1371/journal.pgen.1003745

**Published:** 2013-08-15

**Authors:** Stephanie Sprowl, Marian L. Waterman

**Affiliations:** Department of Microbiology and Molecular Genetics, University of California, Irvine, Irvine, California, United States of America; Cincinnati Children's Hospital Medical Center, United States of America

**Figure 1:**
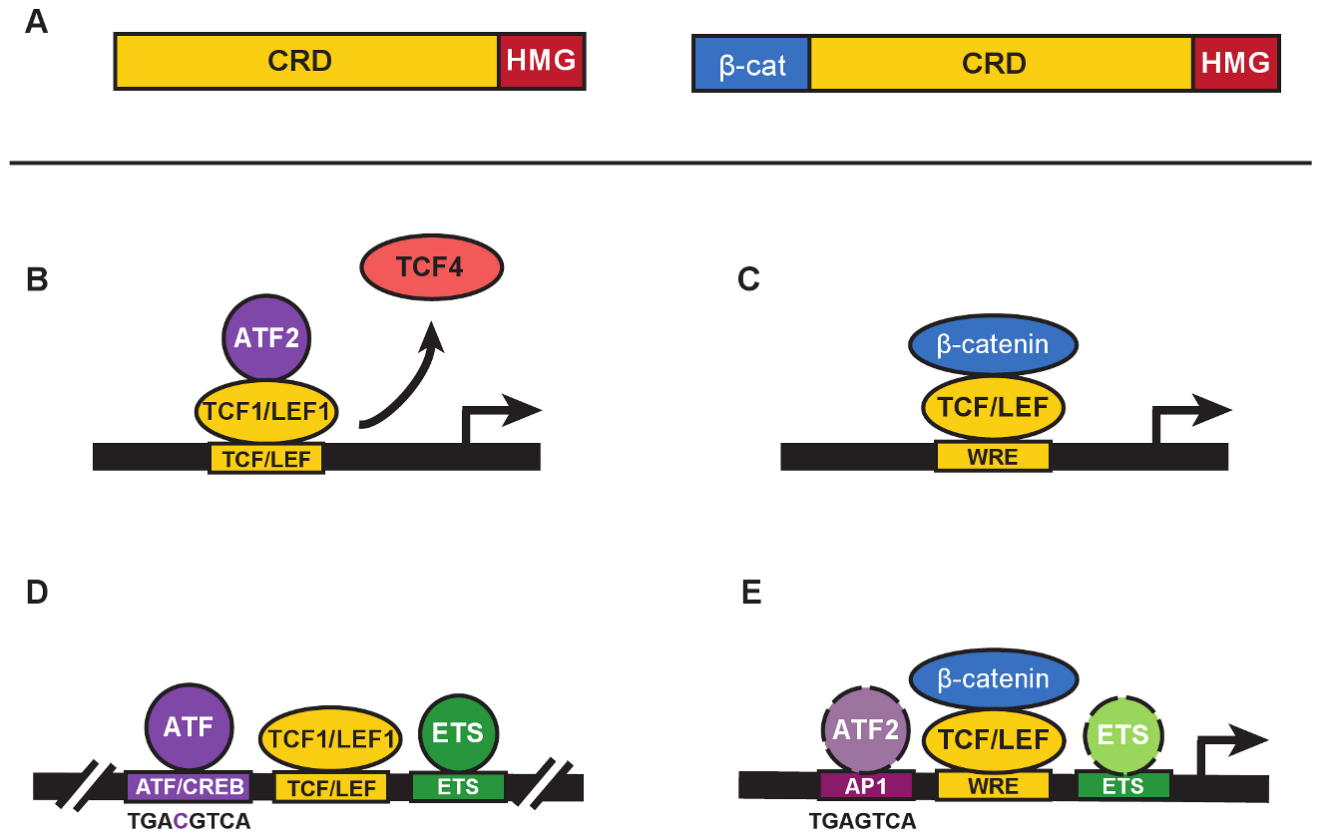
β-catenin–independent and –dependent modes of Wnt signaling. **A.** The general domain structure of TCF/LEF proteins includes a highly variable Context Regulatory Domain (CRD) and the well-conserved N-terminal β-catenin–binding (β-cat) and High Mobility Group (“HMG”) DNA-binding domains. N-terminal truncated forms of TCF/LEFs (left) are naturally occurring and commonly referred to as dominant negatives (e.g., dnTCF1, dnLEF1, etc.) because they block gene regulation by displacing full-length proteins from target genes (right). In the Grumolato study, dnTCF1 and dnLEF1 functioned perfectly well to activate a Wnt reporter gene. **B.** A simplified representation of ATF2 recruitment by TCF1 to activate transcription in a β-catenin–independent manner (referred to as a β-catenin–independent Wnt signaling pathway in Grumolato et al. [13]). In some contexts, displacement of weaker TCF activators such as TCF4 might also contribute to activation. **C.** β-catenin–dependent Wnt signaling requires the recruitment of β-catenin by TCF/LEFs to a Wnt Response Element (WRE) for transcriptional activation of target genes. **D.** A distal enhancer for the T-Cell Receptor alpha chain gene, identified as one of the very first targets for TCF/LEF binding (see references in text), contains closely juxtaposed binding sites for ATF/CREB proteins (consensus binding sequence shown below), LEF/TCFs, and ETS proteins. **E.** ChIP-seq studies of TCF and β-catenin genome-wide occupancy identify significant colocalization of binding motifs for AP1 and ETS transcription factors (see text for references). Consensus sequences for AP1 and ATF/CREB sites differ by a single nucleotide (see panel D for comparison), and ATF proteins are known to bind AP1 sites. Colocalized motifs suggest there is potential for interaction and cooperative crosstalk between β-catenin–bound TCF/LEFs, and ATF/CREB and ETS proteins.

TCF/LEF transcription factors are best known for their role as mediators of Wnt signaling, helping Wnt direct developmental transitions of stem cells in tissues or driving cell transformation and cancer when Wnt is aberrantly active. These factors possess a High Mobility Group DNA-binding domain that recognizes a motif called the Wnt Response Element (WRE: 5′-CTTTGWW-3′) and an N-terminal domain that binds β-catenin (Figure 1A). β-catenin is the cytoplasmic-nuclear mediator that communicates Wnt signals from the plasma membrane to TCF/LEFs for transcription activation (Figure 1C). The vast majority of published studies about TCF/LEFs focus on their recruitment of this mediator to Wnt target genes. This implies that “life” for TCF/LEFs began in 1996 when yeast two hybrid screens identified their mutual, strong interaction [1]–[3]. In fact, discovery of TCF/LEFs had nothing to do with Wnt and β-catenin. TCF/LEFs were first described as DNA-binding proteins that regulated transcription of lymphocyte-specific genes such as the T-Cell Receptor complex, and they did so by cooperating with transcription factors bound to juxtaposed elements in enhancers [4]–[7]. The original TCF1 and LEF1 were each characterized as a set of protein isoforms, differing by the presence or absence of N-terminal (β-catenin) and C-terminal domains, none of which were needed for enhancer activity [8]–[12]. In this issue, Grumolato et al. [13] report that TCF1 and LEF1 have constitutive, elevated activity in leukemia and lymphoma cells. They report that this activity is independent of β-catenin and instead involves direct recruitment of ATF2 and related family members (Figure 1B).

Grumolato et al. describe how the TOPflash reporter for Wnt signaling, a luciferase gene driven by a minimal promoter with multimers of WREs, has elevated constitutive activity in leukemia cell lines. One might assume that this activity derives from TCF/LEF recruitment of β-catenin. However, very little stabilized β-catenin could be detected and reporter activity was recapitulated using truncated forms of TCF1 missing the N-terminal β-catenin–binding domain (isoforms labelled dominant negatives, or dnTCF/dnLEF [Figure 1A]). In another twist, family member TCF4 could not substitute even though it has a β-catenin–binding domain. This meant that selective action by LEF1 and TCF1 occurred through recruitment of other transcription factors via domains distinct from the β-catenin–binding domain. Using a candidate approach, the authors tested for functional interactions with proteins that bind AP1 sites. AP1 factors are homo- and heterodimerizing leucine zipper proteins of the Jun, Fos, ATF, and JDP families [14]. Grumolato et al. report that ATF family members (especially ATF2) bind directly to TCF1 and LEF1, not TCF4, and that interactions primarily require the Context Dependent Regulatory domain (CRD; Figure 1A, B). That TCF1/LEF1-ATF2 interactions are detected in multiple types of hematopoietic tumor cell lines suggests that ATF recruitment might account for a significant portion of the “Wnt reporter activity” in these cell types. Knockdown of ATF2 reduced cell growth and lowered expression of TCF1 and LEF1 target genes, similar to effects from overexpression of a dominant negative form of TCF4. Observations such as these suggest that ATF2 is integral to the regulatory role that TCF1/LEF1 play in lymphocytes.

These discoveries highlight how TCF1/LEF1 are closely intertwined with ATF proteins. Indeed, one of the first interactions for LEF1 and, later, TCF1 was with proteins that bind an ATF/CREB element in the T-Cell Receptor alpha chain enhancer (Figure 1D; [4], [5]); interestingly, ATF4 was first discovered on the basis of its binding to this element (reviewed in [15]). Additional lymphocyte-specific enhancers were discovered as collections of ATF/CREB, TCF/LEF, and ETS elements [15]), and functional studies showed that TCF1 and LEF1 cooperated with these proteins bound to neighboring elements to create strong enhancers. Importantly, the β-catenin–binding domain was entirely dispensable, its deletion enabling even greater activity in some assays [9], [12]. Instead, it was the CRD and a strong DNA-bending function of the HMG domain that was of primary importance; DNA bending enabling a three-way, CRD-dependent interaction between TCF/LEFs and other enhancer factors [16]. The exact identities of the ATF/CREB proteins were unknown and were never fully explored. The Grumolato study brings the past back to the present by identifying specific ATF interactors for TCF1 and LEF1 for the first time, and by showing that immune system cancers possess elevated, functional interactions. The current study does not highlight the DNA binding of ATF2 because it appears to be recruited by TCF1/LEF1 to the Wnt reporters in a protein–protein interaction mode.

An emerging feature of TCF/LEFs that connects with these findings is a growing recognition of a functional split in the vertebrate family. That is, an increasing number of reports show that TCF4 and a fourth family member, TCF3, function as repressors, or at best, weak activators. More and more frequently it seems that TCF1 and LEF1 operate by opposing TCF3/TCF4 repression and providing strong activation. Since TCF1 and LEF1 are strongly active for ATF2 engagement and cooperation, and TCF4 is not, ATFs could be important players in the push-and-pull between family members. The first study to highlight a split in the family used morpholino knockdown and rescue experiments in *Xenopus* embryos [17]. Grumolato and colleagues use the same *Xenopus* system to show that overexpression of dominant negative TCF1 (dnTCF1), but not TCF4, causes axis duplication—an activity attributed to overactive Wnt signaling. It could be, as the authors posit, that dnTCF1 was recruiting ATF proteins to WREs for gene activation. But it is also possible that dnTCF1 was displacing endogenous, repressive TCFs such as TCF3 and/or TCF4 (Figure 1B). Of course, both mechanisms could be involved, but further studies are definitely warranted.

This study raises other questions about TCF/LEFs and β-catenin–independent activation of transcription. Is TOPflash the reliable indicator of Wnt signaling that its common use implies? Or can factors such as ATF2 be recruited to activate this reporter independent of β-catenin? The authors provide a “yes” to the latter question in their system, but a general answer would be best addressed with strategies that avoid overexpression of transcription factors. How much do dominant negative TCF/LEFs contribute to gene regulation? While an exact answer is not known, it is interesting to point out that these forms are expressed at significant levels in lymphocytes [8], [10]. In fact, the discovery of TCF1 came from a T lymphocyte cDNA screen in which all clones were missing the β-catenin–binding domain (the N-terminus–encoding exon discovered years later upon inspection of genomic sequences [6], [8]). Thus, even though definitive connections between β-catenin, TCF1, and LEF1 are clear in lymphocytes (reviewed in [18], [19]), the discordance between their knockout phenotypes should encourage a revisit of this issue. What about cancer? The authors point out the finding in human sebaceous tumors in which mutations have disabled the β-catenin–binding domain of LEF1. This mutation is proposed to be oncogenic because overexpression of dnLEF1 in mouse skin recapitulates sebaceous tumor development [20], [21]. Perhaps β-catenin–independent actions of TCF/LEFs are more prevalent and powerful than currently assumed. Is the ATF/CREB and TCF/LEF interaction common? The tentative answer is yes, because almost every ChIP-seq study of TCF/LEF binding in cancer genomes, and one study of β-catenin–binding to the colon cancer genome, has identified the closely related, ATF-friendly, AP1 response element as a top, cosegregating motif (Figure 1E; [22]–[25], [26]). Several studies also define cosegregating ETS elements [22], [24], [25]. Going forward, it will be important to probe how broadly ATF2 and family members crosstalk to TCF/LEFs (and perhaps β-catenin), and determine what the functional consequences of that crosstalk are in terms of gene programs and cell phenotypes.

*In this Perspective, we use the protein names to refer to TCF/LEFs (i.e., LEF1, TCF1, TCF3, and TCF4). This matches the Grumolato et al. study and makes for logical reading. However, the gene names for TCF/LEFs are different: TCF1 is encoded by the* TCF7 *gene, and TCF3 and TCF4 by the* TCF7L1 *and* TCF7L2 *genes, respectively. LEF1 is the only respite. It is encoded by the* LEF1 *gene*.
